# Metal-Organic Frameworks: Versatile Platforms for Biomedical Innovations

**DOI:** 10.3390/ma16186143

**Published:** 2023-09-09

**Authors:** Cătălin Păun, Ludmila Motelică, Denisa Ficai, Anton Ficai, Ecaterina Andronescu

**Affiliations:** 1 Department of Science and Engineering of Oxide Materials and Nanomaterials, Faculty of Chemical Engineering and Biotechnologies, University Politehnica of Bucharest, Gh. Polizu 1-7, 011061 Bucharest, Romania; 2 Department of Inorganic Chemistry, Physical Chemistry and Electrochemistry, Faculty of Applied Chemistry and Materials Science, University Politehnica of Bucharest, Gh. Polizu 1-7, 050054 Bucharest, Romania; 3 Academy of Romanian Scientists, Ilfov St. 3, 050054 Bucharest, Romania

**Keywords:** metal-organic framework, drug delivery, biomolecule recognition, sensing

## Abstract

This review article explores the multiple applications and potential of metal-organic frameworks (MOFs) in the biomedical field. With their highly versatile and tunable properties, MOFs present many possibilities, including drug delivery, biomolecule recognition, biosensors, and immunotherapy. Their crystal structure allows precise tuning, with the ligand typology and metal geometry playing critical roles. MOFs’ ability to encapsulate drugs and exhibit pH-triggered release makes them ideal candidates for precision medicine, including cancer treatment. They are also potential gene carriers for genetic disorders and have been used in biosensors and as contrast agents for magnetic resonance imaging. Despite the complexities encountered in modulating properties and interactions with biological systems, further research on MOFs is imperative. The primary focus of this review is to provide a comprehensive examination of MOFs in these applications, highlighting the current achievements and complexities encountered. Such efforts will uncover their untapped potential in creating innovative tools for biomedical applications, emphasizing the need to invest in the continued exploration of this promising field.

## 1. Introduction

Metal-Organic Frameworks (MOFs) have garnered significant research attention for their tunability and high surface area, rendering them highly promising for various applications. 

According to Awasthi et al. [1], the crystal structure of MOFs allows for the precise tuning of their properties by adjusting the metal ions and ligands used in their synthesis. 

**Figure 1:**
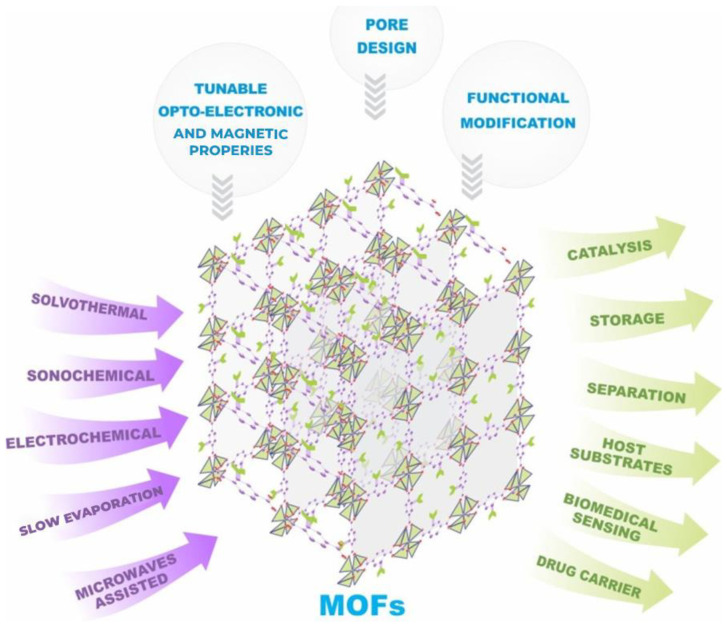
A multitude of synthetic routes along with the possibility of structural tuning for a wide range of applicability of MOFs to obtain compounds with desired properties.

MOFs’ hybrid structure and pore size control make them capable of exhibiting a large area of applicability with a high specific surface area. This versatility enables diverse applications in gas storage, catalysis, purification, detection, separation of hazardous compounds from the environment, energy, and biomedical applications. Several studies, including Awasthi et al. [1] and Hu et al. [2], have demonstrated the ability to fine-tune MOFs’ properties for specific applications. Figure 1 aims to highlight these points, showing that MOFs exhibit a large area of applicability, which researchers have reported across various synthesis routes, making it one of their most significant advantages.

**Figure 2:**
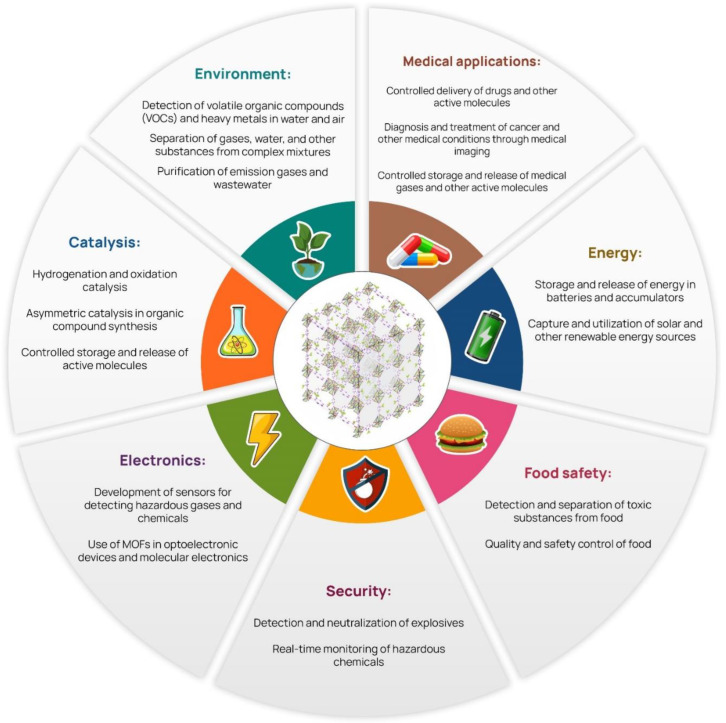
Areas of interest for the study of MOFs.

A comprehensive overview of the diverse research landscape associated with MOFs, outlining various areas where their study holds significant implications, can be found in Figure 2.

Using data sheets from CD Bioparticles, Table 1 details the properties of some of the MOFs they produce, and it is clear how these compounds exhibit physicochemical characteristics suitable for drug delivery, including examples of the biomedical potential of MOFs in drug delivery. Just by comparing them with other porous materials (especially other micro or mesoporous materials), it can be observed that the specific surface area can reach 3000 + m^2^/g; the pores can be from the microporous range or the mesoporous range (over 3 nm). The window size is also defined as the size of the molecules that can pass through these pores, and this range is between 0.34 and <2 nm, which is well suited to the size of the small drugs. The examples retained in Table 1 are selected to cover some of the most important applications in the biomedical field. 

Beyond drug delivery, MOFs have been successfully utilized in biomedical applications such as sensing, biomolecule recognition, biosensors for disease diagnosis, and immunotherapy. The versatility of MOFs in these applications is due to their ability to form complex three-dimensional network structures that can accommodate various molecules, including drugs, DNA probes, and RNA-based therapeutics. The studies conducted by Gao et al. [16] and Adeel et al. [17] demonstrate the successful utilization of MOFs in sensing various biomolecules, including glucose and cholesterol, for diabetes management. MOFs have also been used to recognize and separate biomolecules, making them a promising candidate for the separation and purification of biomolecules, as shown by Hosando et al. [18].

The study by Nunzio et al. [19] explored the use of MOFs in photochemical sensing applications. It highlighted the role of the ligand typology in the complexity of the structures formed. Studying MOFs for biomedical applications represents a significant challenge due to the need to precisely control their properties and interactions with biological systems. Zhao et al. [20] highlight the considerable challenge of precise control over their proprieties and interactions with biological systems. This need for precision in MOF-based biosensors for disease diagnosis is highlighted by the study conducted by Yin et al. [21], who developed a MOF-based biosensor for detecting Alzheimer’s disease biomarkers exhibiting high sensitivity and selectivity. The potential of MOFs in immunotherapy applications is also supported by the studies conducted by Zhao et al. [22], Ni et al. [23], and He et al. [14], where they reported the development of MOF-based immunotherapeutic agents and MOFs as a delivery system for RNA-based therapeutics, respectively.

MOFs can also be integrated with various transduction techniques, such as optical, electrochemical, and piezoelectric, to detect and quantify specific analytes (Naresh et al.) [24].

**Figure 3:**
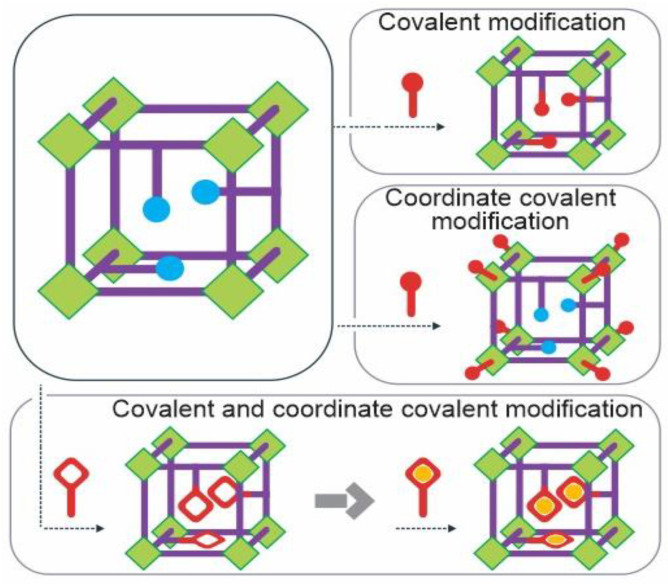
Combination of post-synthetic modification strategies for metal-organic frameworks (covalent, coordinate covalent, or both).

The strategic combination of post-synthetic modification strategies for metal-organic frameworks, as shown in Figure 3, provides insight into how covalent, coordinate covalent, or even a mix of both methods can be employed to achieve the desired characteristics and functionalities in MOFs.

Metal-Organic Frameworks (MOFs) represent versatile platforms for biomedical innovation due to their tunable properties, high surface area, and ability to form complex three-dimensional network structures that can accommodate various molecules. For this purpose, the present review aims to highlight the correspondence of the MOFs’ crystal structure, which is induced by the duality of the metal ion and ligand used in their synthesis. As a consequence of the structural changes induced by the used building block (metal ion and ligand), the properties can be accordingly adjusted, making them suitable for sensing, drug delivery, biomolecule recognition, biosensors for disease diagnosis, and immunotherapy applications. The typology of the ligands used in MOF synthesis also plays an essential role in the complexity of the structures formed. On the other hand, studying MOFs for biomedical applications poses significant challenges, such as precisely controlling their properties and interactions with biological systems. Despite these challenges, MOFs continue to show promise as a platform for innovative biomedical applications, as demonstrated by recent studies on MOFs-based biosensors for disease diagnosis and MOFs-based immunotherapeutic agents.

Consequently, this review is dedicated to examining the link between the structural properties of MOFs, shaped by the employed metal ion and ligand, and their capabilities in various biomedical applications, emphasizing the essential need for sustained research efforts and investment in this highly innovative and promising domain.

## 2. MOFs in Drug Delivery Applications

MOFs have attracted considerable focus in targeted drug delivery due to their unique design, impressive ability to interact with substances, and facility for chemical customization, allowing them to contain drugs within specific internal spaces. These characteristics enable MOFs to hold targeted medications, offering opportunities for controlled release. The meticulous crafting of MOFs permits tailoring drug release patterns, rendering them flexible for diverse medical conditions. Moreover, the skillful adjustment of MOFs’ structure enhances their proficiency in assimilating drugs, making precise delivery to designated tissues or cells possible, as shown in various systems responsive to pH changes. Such qualities of MOFs leverage their capability to heighten drug delivery effectiveness, laying the groundwork for advancements in personalized medicine. The management of drug release is crucial for achieving the best therapeutic results while reducing undesirable side effects.

### 2.1. pH-Responsive MOFs

Several studies have demonstrated the potential of MOFs in controlled drug release. pH-triggered drug release can be realized by pH-dependent carrier disassembly. 

**Figure 4:**
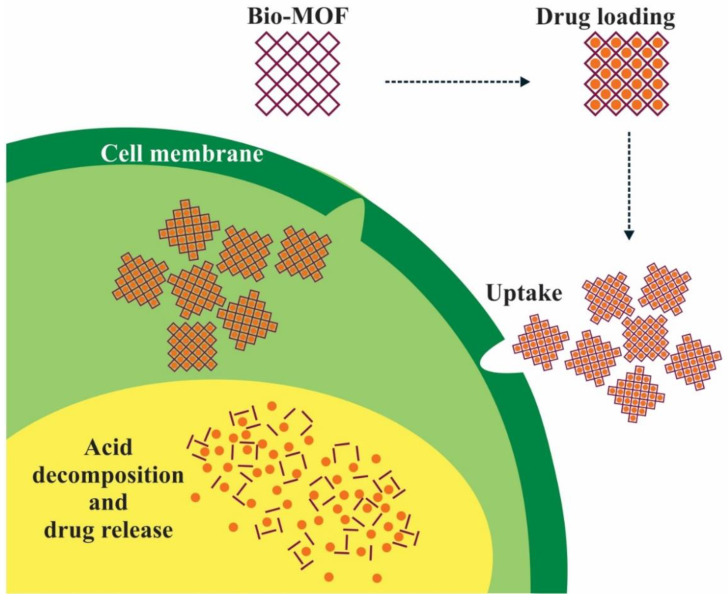
Disassembly mechanism of the pH-dependent stimuli-sensitive MOF carrier.

For example, an acidic environment in tumor tissue makes pH one of the most widely investigated stimuli for targeted and controlled drug release, as presented in Figure 4.

In her research, Jian et al. [25] developed a technique for delivering functional proteins to particular cells using “clickable” zeolitic imidazolate framework-8 (ZIF-8) nanoparticles with potential implications in cancer treatment and precision medicine. This information has been discussed in the research papers of Rahaman et al. [26] and Cai et al. [27], which developed drug delivery systems that respond to specific environmental stimuli, such as pH, to improve drug efficacy and reduce side effects. IRMOF-16 was studied as a potential pH-responsive drug delivery carrier for curcumin and exhibited pH-dependent behavior in the delivery process. Du et al. [28] investigated the Cu-MOFs@Keratin drug delivery system, which demonstrated responsiveness to pH and ROS, with enhanced drug release observed under acidic and oxidative conditions. 

In this study, Muhammad et al. [29] and his team developed a pH-responsive bi-MIL-88B MOF coated with folic acid-conjugated chitosan, serving as an effective nanocarrier for targeted drug delivery of 5-Fluorouracil, showcasing controlled drug release in cancer cells and demonstrating potential for smart drug delivery systems.

Lin et al. [5] synthesized a pH-responsive MOF, MIL-125(Ti), modified with Pluronic F127 and chitosan monomers and loaded with doxorubicin. The carrier was found to be temperature- and pH-responsive, with the potential for use in cancer treatment. ZIF-8 is also widely used in drug delivery due to its easy fabrication process and good biosafety. 

In an extensive investigation by Gongsen Chen et al., [15] the utilization of MOF-5 was studied as a sustained release carrier for the antitumor drug Oridonin. The research revealed that MOF-5 successfully encapsulated ORI, maintaining its intrinsic structure, as shown by FTIR and TG analysis. Importantly, when studying the in vitro release properties of ORI@MOF-5 under various pH conditions, it was evident that the release behavior of the compound was less affected by changes in pH, highlighting its versatility.

### 2.2. Multi-Stimuli Responsive MOFs

Drug delivery systems that respond to multiple stimuli can improve therapeutic efficacy. Liu et al. [30] synthesized a co-delivery system by coordinating 2-methylimidazole, zinc ion, and doxorubicin, followed by the surface decoration of indocyanine green for targeted drug release and thermos-ablation. Trushina et al. [31] combined UiO-66 nanoparticles with mesoporous SiO_2_ and folate-conjugated pluronic F127 to prepare a core-shell MOF@SiO_2_/F127-FA drug delivery carrier for targeted cellular uptake in cancer treatment. Silica coating enabled the grafting of target molecules to the surface and improved stability, while further modification with pluronic and folic acid improved biocompatibility and targeting. The DOX-loaded UiO-66@SiO_2_/F127-FA nanoparticles were evaluated for properties and antitumor activity, demonstrating potential for small molecule delivery and increasing the practical value of MOFs. Safnejad et al. [32] synthesized a non-toxic La-based MOF with 3,4-di hydroxycinnamic acid as a linker, which exhibited good biocompatibility with human breast cancer cell lines and confirmed the ability of 3,4-DHCA to treat the cells. With promising results, Li et al. [20] investigated a Sr-based MOF as a ketoprofen carrier for osteoarthritis treatment.

### 2.3. MOFs in Enhancing Drug Solubility and Bioavailability

MOFs can also improve the solubility and bioavailability of poorly soluble drugs. Yan et al. [33] developed a “prodrug-ZIF-8” strategy for the targeted delivery of doxorubicin to solid tumors. Ma and Zhang [34] synthesized a redox-responsive paclitaxel drug delivery platform using ZIF-8 as the vehicle, cystamine as the linker, and redox-sensitive material. They found that higher glutathione concentration and lower pH favored releasing paclitaxel from ZIF-8/cystamine/paclitaxel, resulting in a higher tumor-killing effect than the free paclitaxel solution. Sun et al. [35] developed a hyaluronic acid-targeted and pH-responsive drug delivery system based on ZIF-8, which encapsulated D-α-Tocopherol succinate in ZIF-8 compounds and coated them with a hyaluronic acid shell, achieving a tumor-specific and on-demand drug delivery system that improved the treatment efficiency. 

**Figure 5:**
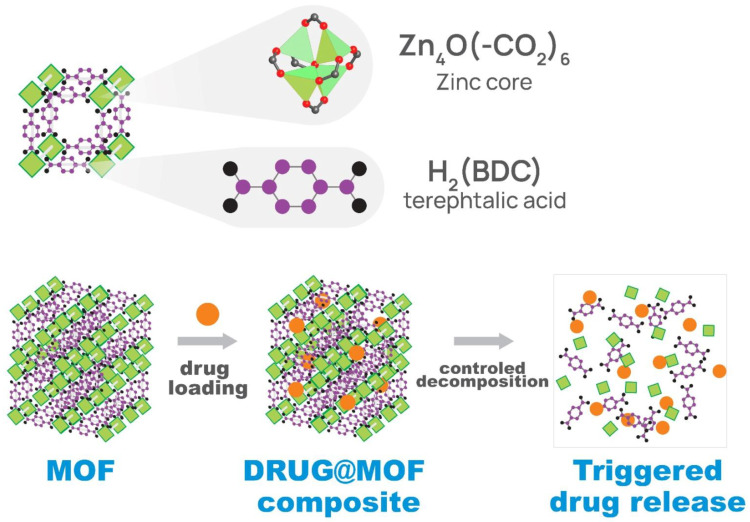
Schematic representation of drug encapsulation within MOF-5, leading to controlled and triggered drug release.

Suresh et al. [36] highlighted a novel technique to enhance the dissolution and solubility of hydrophobic drugs, specifically curcumin, sulindac, and triamterene. They encapsulated these drug molecules within water-reactive MOFs, prominently MOF-5. This approach prevented the crystallization of the drug in its amorphous state and initiated its immediate release when the MOF underwent hydrolytic decomposition. As illustrated in Figure 5, this method offers a strategic solution for drugs with poor solubility. It addresses the typical concerns associated with amorphous drug delivery, such as the amorphous form’s physical instability.

### 2.4. MOFs as Gene Carriers

MOFs have shown promising results as gene carriers for treating genetic disorders and certain types of cancer due to their tunable porosity, biocompatibility, and ease of surface functionalization. However, efficiently and safely delivering therapeutic genes to target cells remains a significant challenge. For instance, Chang Liu et al. [37] synthesized a biocompatible MOF carrier, ZIF-8, for the efficient delivery and remote regulation of CRISPR-Cas9 ribonucleic acid protein (RNP)-based gene editing.

To summarize, MOFs have risen to prominence in drug delivery due to their ability to be precisely engineered, extraordinary capacity to absorb substances, and unique way of encapsulating drugs within their cavities. Controlled drug release is crucial for optimal therapeutic outcomes, and MOFs have demonstrated outstanding potential. pH-triggered drug release is a widely investigated stimulus for targeted and controlled drug release, particularly in tumor tissue, because many cancers induce acidic conditions. Thus, this stimulus can be suitable for targeted delivery within the tumor tissue. Non-toxic La-based MOFs, such as MIL-101(Cr) and UiO-66, were primarily studied for the intracellular delivery of doxorubicin to cancer cells.

Furthermore, MOFs can improve the solubility and bioavailability of poorly soluble drugs. Overall, MOFs have shown great promise in drug delivery and have the potential to revolutionize medicine due to their versatility and ability to respond to various stimuli. 

## 3. Exploring the Potential of MOFs in Biomolecule Recognition and Sensing

### 3.1. MOF Structure and Properties for Biomolecule Recognition 

Thanks to their adjustable porous structure, metal-organic frameworks (MOFs) have significant potential for recognizing and separating biomolecules. This adaptability enables the creation of MOFs with particular characteristics that are perfect for such uses, a point emphasized by Hassanpour and colleagues [38]. Furthermore, MOFs exhibit an extensive capacity for interaction, contributing to their marked efficiency when utilized as materials in nanomedicine, a finding noted by Pashazadeh et al. [39]. The structural design of MOFs is vital to their ability to recognize specific molecules since their porous nature allows them to house various recognition elements and ligands that can attach to the target biomolecules, a feature underlined by Feng and associates [40]. 

**Figure 6:**
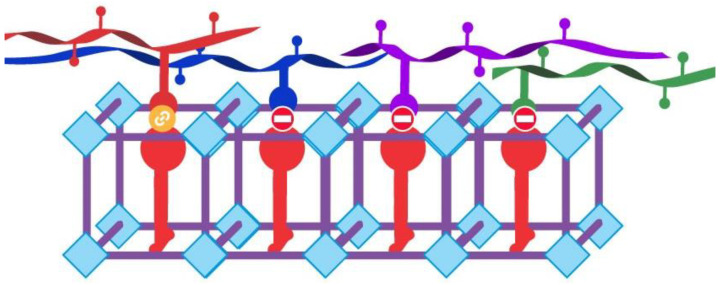
Schematic representation. Selective molecular capture by MOF structures.

Leveraging its tunable structure, Figure 6 exemplifies the challenge of effectively designing a MOF. By strategically functionalizing MOFs with specific groups, they can selectively recognize certain molecules while bypassing others, forming the cornerstone of selective recognition and separation.

#### MOFs in the Selective Recognition and Sensing of Biomolecules

The choice of ligand and ion metal has to be synergistically carried out because the properties of these structured materials depend on and thus affect the selectivity and sensitivity of the final biosensor.

By functionalizing MOFs with specific groups, they can selectively recognize certain molecules while bypassing others. This customization is made possible by the essential synergy between the ligand and the metal ion, which influences the material’s properties and consequently affects the selectivity and sensitivity of the resulting biosensor. This selective recognition and separation is a complex goal, but can be accomplished with careful control over the MOF’s structure. A MOF based on Cobalt (II) was developed as a significant example of this process. This particular MOF showed an outstanding ability to detect the vitamin B12 biomarker, methylmalonic acid (MMA), through a fluorescence turn-on response capability [41].

Biomolecule selective recognition was achieved using a tetratomic phosphonate ligand (H8L)-based MnII-MOF (H8L-Mn-MOF) developed by Chakraborty et al. [42]. The MOF demonstrated demanding “Turn-On” behavior towards L-arginine (Arg) over L-lysine (Lys) and other amino acids, with a detection limit of 7.1 ppm in an aqueous medium. The material also showed similar responsive behavior in different bio-fluids, such as fetal bovine serum and human urine, suggesting its bio-applicability.

### 3.2. Computational and Experimental Approaches in MOF Recognition Studies

Recent studies have employed both computational and experimental techniques to explore interactions with MOFs. Mostafavi et al. [43] utilized dispersion-corrected Density Functional Theory (DFT-D3) to investigate the interactions between glycine amino acid and MOF-5, identifying substantial connections between them. This intense interaction energy, coupled with evidence that glycine forms chemical bonds with MOF-5, paves the way for developing nano-scaled drug delivery systems. This research contributes to the broader understanding of how biomolecules interact with nanostructures to create functional materials. 

In a parallel development, Liao et al. [44] crafted a novel immunoprotein by integrating a Nanobody (Nb) with a biomimetic mineralized MOF. This integration overcame existing challenges in immunoassay sensitivity. By encapsulating succinylated horseradish peroxidase (sHRP) within a single MOF structure, they improved the electro-sensing of aflatoxin B1 (AFB1). These investigations, harnessing computational approaches such as DFT-D3 and experimental innovation in MOF-based electrochemical sensors, demonstrate the depth and versatility of MOF research in biomolecule recognition. Collectively, they underscore the vital role of combined computational and experimental methodologies in unlocking MOFs’ potential for recognizing and interacting with biomolecules.

### 3.3. MOFs in Diagnostic Applications for Disease Monitoring and Enzyme Immobilization

The structure of MOFs offers novel strategies for enzyme immobilization. Shortall et al. [45] investigated the stability of MOFs when used as enzyme supports. Their findings emphasized the importance of selecting the right buffer-MOF combinations and suggested that coatings, notably polyacrylic acid (PAA), further enhance stability. 

A central aspect of MOFs’ enzyme incorporation lies in their capacity to co-encapsulate multiple enzymes within their framework. Enzymes can be trapped within pores, anchored to surfaces, benefit from an enhanced microenvironment, or be incorporated through in situ encapsulation during synthesis [46].

**Figure 7:**
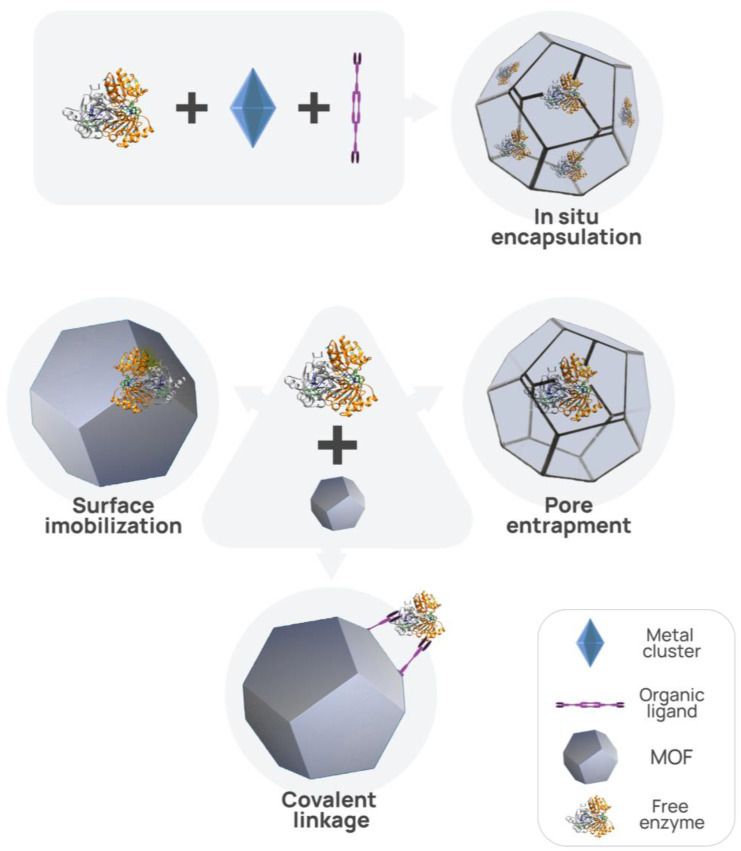
Schematic representation of different immobilizations of enzymes on/in MOF.

These methods can be seen in Figure 7, illustrating the different ways enzymes are attached to the MOFs.

MOFs have proven very challenging in detecting and separating molecules in the diagnostic realm, especially in diagnosing conditions such as neurological disorders and epilepsy. The work of Zhang et al. [47] illustrates this versatility, where they co-encapsulated multienzyme systems in MOFs for the specific detection of lactate.

Significant improvements in current response have been observed when using 2D metal-organic layers (MOLs) to support redox enzymes, as shown by Xiong et al. [47]. MOFs functionalized with specific antigens have also been used to detect antibodies, such as anti-HIV antibodies. 

Further advances include aptasensors for antibiotics such as kanamycin [48] and platforms specifically for detecting uric acid, highlighted in recent research by Yang et al. [49]. These examples underline the breadth of applications MOFs can offer in diagnostics.

Recent advancements extend to creating a lanthanide metal-organic framework capable of the selective optical detection of Favipiravir, a medication used to treat COVID-19. This specific MOF exhibits high detection sensitivity and recyclability, according to the study by Wang et al. [50]. 

The use of MOFs in diagnostics has been demonstrated through their successful identification of substances such as ST and 5-HI-3-AA in human serum and urine, as reported by Zhong et al. [51]. This accomplishment in a separate line of investigation underscores the broader potential of MOFs in detecting and tracking biomolecules.

### 3.4. MOFs in Biosensing and Biotechnological Innovations

The possibilities for MOFs in the fields of biosensing and biotechnology are extensive. Their unique structure and ample interactive capacity facilitate the efficient recognition of proteins and peptides, as observed by Cedru et al. [52]. Furthermore, MOFs serve as foundations for securing enzymes, as noted by Souza et al., and offer a basis for supporting nano-zymes and dual-mode sensing techniques, according to Yu et al. [53], opening up new paths for biosensing applications. 

Pillararene-incorporated MOFs have been recognized for their potential in supramolecular recognition and selective separation, adding another layer to the biosensing capabilities of these materials (Wu et al.) [54].

In addition to the abovementioned applications, MOFs have shown potential in biomolecule sensing for fields such as radiology, radiotherapy, and immunology. MOFs have been explored as contrast agents for magnetic resonance imaging (MRI). Bunzen et al. [55] explored their potential and grouped three types of materials that can be used: MRI-active MOFs, composite materials based on MOFs, and MRI-active compounds loaded into MOFs. 

### 3.5. MOFs for Cardiac and Cancer Biomarker Detection

MOFs have been instrumental in detecting cardiac biomarkers, including specific proteins such as troponin T. Saeidi et al. [56] utilized MOFs to develop a multilayer nanostructure, enabling the accurate and reliable detection of troponin T, a key indicator of heart injury. The sensitivity and specificity of MOFs could revolutionize the diagnostics of cardiac conditions.

Cancer detection and monitoring have also benefited from MOFs. Du et al. [57] developed a highly efficient impedance biosensor for cancer cell detection using folic acid-functionalized zirconium MOFs. This innovation represents a significant step forward in cancer diagnostics, offering a powerful tool to detect cancerous cells in various stages. MOFs have been further applied to stimuli-responsive drug delivery systems, including pH, temperature, or light irradiation-triggered drug release, enhancing targeted therapy.

### 3.6. MOFs in Electrochemistry, Small Molecule Sensing, and Mycotoxin Detection

MOFs are potential electrode modifiers for the electrochemical detection of small molecules such as epinine and venlafaxine. As electrode modifiers, MOFs (Dourandish et al.) [58] provide platforms for the co-detection of biomolecules, significantly advancing the field of electrochemical biosensing. These frameworks’ well-defined structures and selective sensing mechanisms enable detailed computational investigations, shedding light on the selective sensing of potential therapeutic compounds. Furthermore, MOFs have been harnessed for the selective detection of mycotoxins, potent toxic compounds produced by fungi. The utilization of luminescent MOF (LMOF-241) by Hu et al. [59] exemplifies the sensitivity and selectivity of MOFs in recognizing mycotoxins, offering a critical approach to monitoring and controlling their presence in food products.

## 4. MOFs in Biosensing: Challenges and Opportunities for Disease Diagnosis and Monitoring

**Figure 8:**
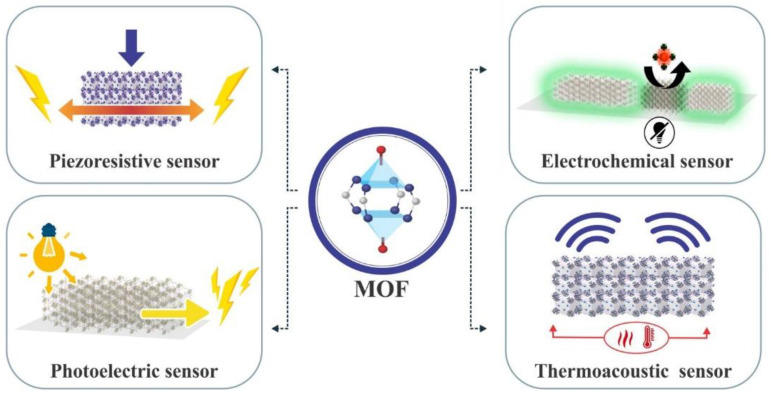
Sensors named after their physical properties.

MOFs have gained interest as potential biosensors for disease diagnosis and monitoring due to their ability to integrate with various transduction techniques, such as optical, electrochemical, and piezoelectric methods. In Figure 8, sensors are named after their physical properties, showcasing the categorization based on characteristics. 

### 4.1. Applications of MOFs in Disease Detection

Recent studies have explored using MOFs in biosensors for diagnosing and monitoring diseases such as epileptic seizures, myocardial infarction, and Parkinson’s disease. For instance, a dual-range lactate oxidase-based screen-printed amperometric biosensor using Cu-MOF cross-linking has been developed to analyze lactate in diversified samples, including sweat, saliva, and wine, demonstrating the potential of MOFs as biosensors, as shown by Silva et al. [60]. Gupta et al. [61] developed an impedimetric sensor for the cardiac marker troponin I (cTnI) using a composite of copper-MOF with polyaniline, highlighting the potential of MOFs in developing biosensors for the diagnosis and monitoring of various diseases. Yan et al. [62] created a “Signal-on” ECL immuno-sensor for detecting cTnI using functional nanoflakes called RuMOFNSs. 

Another biosensor developed by Zhong et al. [51] was utilized to create a stable, water-soluble fluorescent biosensor for detecting serotonin (ST) and its metabolite 5-hydroxy indole-3-acetic acid (5-HI-3-AA). Moreover, a novel layered fluorescent metal-organic nanomaterial with a honeycomb topology based on europium, [Eu(pzdc)(Hpzdc)(H2O)]n (ECP), has been synthesized by Moghzia et al. [63], and exhibits fast response and high selectivity for dopamine detection in clinical samples. A different approach was taken by Xie et al. [64], who reported the development of a highly selective and sensitive label-free MOFs-based fluorescent sensor for detecting dopamine in urine samples from Parkinson’s patients. These biosensors demonstrate the potential of MOFs in various detection techniques, which could be used to diagnose and monitor multiple diseases.

### 4.2. Development of Electro-Chemiluminescent (ECL) Biosensors

In addition to the biosensors discussed earlier, electro-chemiluminescent (ECL) biosensors have also gained attention due to their high sensitivity. Lv et al. [65] created ECL biosensors using aggregation-induced emission (AIE) probes for bioanalytical detection, which offers more choices for improving ECL sensors.

Wang et al. [66] reported the creation of ECL biosensors using multifunctional MOFs (Au@Co-MOF@ABEI) and 3D magnetic walking nanomachines for the ultrasensitive detection of Burkholderia pseudo-mallei, a pathogen that causes melioidosis. The nanocubes immobilized ABEI and exhibited peroxidase-like activity to produce reactive oxygen species, enhancing ECL signals. The biosensor could detect the pathogenic gene down to 60.3 aM and B. pseudo-mallei down to 9.0 CFU mL^−1^ in serum samples. This work provided a promising tool for early diagnosis and disease surveillance.

### 4.3. Advanced Sensor Techniques and Their Applications

Recently, a ratiometric fluorescent sensor based on NH_2_-MIL-101(Fe) with molecular imprinting has been developed by Wang et al. [67], which exhibits outstanding sensitivity, selectivity, and super anti-interference ability, and can be used for precise and rapid detection of domoic acid. This neurotoxin affects the central nervous system.

A type of electrochemical sensor for the detection of dopamine, an essential neurotransmitter of the nervous system, was developed by Ji et al. [68], utilizing the electrochemical sensing potential of Cu-TCPP frameworks and the conductivity of graphene nanosheets, resulting in an ultrasensitive and straightforward detection method with a detection limit of 3.6 nM in the first linear range. The sensor shows potential for use in diagnosing and monitoring neurological diseases.

### 4.4. Wearable and Self-Powered Sensors

According to Rayegani et al. [69], recent developments in wearable sensor technology have created battery-free and self-powered devices for continuous and accurate monitoring of various medical signals in the human body. These smart wearable sensors utilize triboelectric nanogenerators (TENG), piezoelectric nanogenerators (PENG), and hybrid nanogenerators that combine the abovementioned, highlighting the potential of MOFs in creating self-powered sensors for health monitoring. These sensors have a variety of purposes, structural designs, and electric performances and are crucial for health monitoring, including older adults, patients with unique conditions, and those recovering from illness. This new technology offers potential for the analysis of long-term bodily movement status.

Developing efficient and sensitive sensors for detecting hydrogen peroxide (H_2_O_2_) in biological systems is significant for the early diagnosis and treatment of tumors. Recent studies by Xuelian et al. [70] have demonstrated the potential of metal-organic frameworks (MOFs) as a platform for sensitive H_2_O_2_ detection and tumor cell inhibition. MIL-47(V)-OH was shown to convert H_2_O_2_ into ·O_2_^−^ (anionic radical) and inhibit tumor cells, making it a promising material for H_2_O_2_ detection and application in biological diagnoses and oncology therapy. Similarly, the study by Yang Li et al. [71] showed that BODIPY@Eu-MOF demonstrated excellent fluorescent detecting performances for H_2_O_2_ detection and F^-^ and glucose detection in living cells, indicating the potential of MOFs as a multifunctional platform for biological sensing applications.

### 4.5. Challenges and Future Perspectives

Despite the potential of MOFs for biomedical applications, achieving precise control over the reaction conditions during synthesis and minimizing the presence of impurities or defects in the final product is a significant hurdle. Researchers have investigated novel synthetic approaches to control MOF growth, such as microwave-assisted synthesis and utilizing templates or scaffolds. Another obstacle pertains to the stability of MOFs in biological environments, particularly when exposed to enzymes or other biomolecules. Scientists have been exploring post-synthesis modifications, such as surface functionalization with polymers or peptides, to bolster the stability and biocompatibility of MOFs.

The development of self-powered wearable sensors utilizing MOFs holds great potential for advancing healthcare technology and facilitating the continuous and long-term monitoring of individuals’ health status. Further research and development efforts in this domain could address the challenges associated with MOF synthesis, stability in biological environments, and seamless integration into wearable devices. This progress could lead to practical and effective healthcare monitoring and diagnostics solutions.

## 5. Exploring the Potential of MOFs in Biomedical Applications beyond Sensing and Biosensors

### 5.1. Catalysis and Biomedical Chemistry

Copper-based MOFs have been shown by Singh et al. [72] to benefit biomedical applications beyond sensing and biosensors by serving as efficient and regioselective catalysts in click reactions. These MOFs offer high porosity, recyclability, and unusual catalytic activity. Simms et al. [73] also proposed a comprehensive analytic and analytical perspective of MOFs as efficient catalysts in biomedical applications, advancing research in the field. Their study provides molecular insights into MOFs’ potential as nano-zymes for hydrolysis reactions.

Zhu et al. [74] demonstrated that MOFs play an essential role in biochemistry by providing a platform for efficient catalysis and molecular-level insights, and this was evident through their study of the active site behavior of Ru@MIL-101(Cr) catalysts in alcohol conversion, which revealed that MOFs serve as efficient supports for the catalytic process. Saleh et al. [75] demonstrated the efficient synthesis of a novel Yttrium-Metal-Organic Framework catalyst using microwave synthesis techniques. The Y-MOF was then evaluated for its potential as a nanocatalyst in synthesizing pyrazolopyranopyrimidine derivatives, showing promising anticancer properties against breast cancer cells. This study contributes to the advancement of biomedical chemistry. It highlights the potential of MOFs as efficient and recyclable catalysts for synthesizing bioactive compounds with potential applications in cancer therapy. It is worth mentioning Melchiorre et al.’s study [76] on the ketalization of glycerol with ethyl levulinate catalyzed by MIL-88A to highlight the importance of research into the development of efficient and sustainable heterogeneous catalysts for the advancement of new technologies in biomedicine, such as drug synthesis or biomaterials production.

### 5.2. Tissue Engineering and Medical Imaging

MOFs have the potential for 3D tissue scaffolds. Kang et al. [77] created an exosome-functionalized PLGA/Mg-GA MOF scaffold to accelerate bone regeneration with enhanced osteogenic, angiogenic, and anti-inflammatory properties. The platform showed outstanding biocompatibility and excellent osteogenic differentiation of hBMSCs. Slowly released exosomes stabilized the bone graft environment, ensured blood supply, and accelerated bone regeneration. This approach offers promise for bone tissue regeneration. Zhuang et al. [78] explored the use of MOFs for developing contrast agents that can provide more precise morphological and pathophysiological details for diagnosing and treating glioblastoma. The potential of iron oxide-, manganese (Mn)-, gadolinium (Gd)-, 19F-, and copper (Cu)-incorporated nanoplatforms for GBM imaging, as well as dual-modal or triple-modal nanoprobes, were discussed as means to overcome the limitations of each imaging modality.

Han et al. [79] developed a two-dimensional MOF, D-ZIF-67, with high oxidase-like activity for glutathione detection. This work provides a simple platform for visual GSH detection and highlights the potential of MOFs as nano-zymes in biomedicine.

### 5.3. Wound Healing and Bacterial Infections

Due to the need for effective skin wound healing treatments, Wang et al. [80] developed a donut-like copper/nicotinic acid MOF and composite hydrogels with superior bio-activity. Their discovery showed that the rough surface of the MOF facilitated the efficient loading and release of primary fibroblast growth factors, promoting angiogenesis and collagen deposition. Bacterial infections, another challenging area of biomedicine, where studying metal-organic compounds begins to take a broader profile, were also partly addressed by Sheta et al. [81]. Their research highlighted the ability of Fe(III)-MOF to inhibit the growth of bacteria, fungi, and yeasts, with excellent antimicrobial efficacy, at a concentration of 50 and 25 μg/mL Fe(III)-MOF. These results pave the way for using these materials as effective and safe antimicrobial agents. Chen et al. [82] reviewed the antibacterial applications of 2D molybdenum sulfide (MoS2) and its derivatives, highlighting their potential to address bacterial infections. They discussed the material’s structural characteristics, antibacterial performance, mechanisms of action, and the challenges and perspectives in the field. However, the review did not specifically focus on MOFs in biomedical applications.

### 5.4. Advanced Technologies in Biomedicine

The study on Fe-BTC by Mannias et al. [83] highlights the potential of MOFs in biomedical applications, specifically in the one-pot immobilization of biomolecules. According to Falahati et al. [84], metal-organic frameworks with nanozyme activity have shown promising results in biomedical applications, particularly in biocompatible nano-/micro-motors, which can improve the motor behaviors in the propulsion function, conductivity, targeting, and possible elimination in cancer therapy. The development of MOF-NZs-based nanomotors could address ongoing problems in the field and pave the way for more effective cancer treatment. However, reducing the toxicity of the required propellants remains a challenge.

### 5.5. Immuno-Therapy Interventions

A specific example of MOF application in immuno-therapy is RiMO-301, a hafnium-based MOF. A phase 1 dose-escalation study of RiMO-301 in conjunction with palliative radiation has been conducted in advanced tumors, as highlighted by Koshy et al. [85]. RiMO-301 has been shown to enhance the antitumor effects of ionizing radiation via a novel radiotherapy-radio dynamic therapy mode. This unique action enhances radiotherapy and amplifies the immunotherapeutic effects of immune checkpoint inhibitors. The clinical trial revealed promising signs of efficacy, demonstrating the potential of RiMO-301 as a radio-enhancer. Furthermore, hafnium-based MOF nanoparticles, such as UiO-66-NH2(Hf), are being explored as radiosensitizers to improve radiotherapy efficacy in esophageal cancer by enhancing X-ray absorption, as illustrated by Zhou et al. [86]. By inducing apoptosis in tumor tissues and increasing radiation absorption, MOFs such as UiO-66-NH2(Hf) optimize the overall efficacy of immunotherapy treatments. The strategic application of MOFs in these contexts supports the development of more effective and personalized treatment strategies, marking a significant advancement in immuno-therapy interventions.

## 6. Conclusions and Future Perspectives

Metal-organic frameworks represent versatile platforms for biomedical applications due to their tunable properties, high surface area, and ability to form complex three-dimensional network structures that can accommodate various molecules. MOFs have shown great promise in drug delivery, biomolecule recognition, sensing, biosensors, wound healing, catalysis, tissue engineering, bacterial infections, and medical imaging. In drug delivery applications, MOFs can improve the solubility and bioavailability of poorly soluble drugs, and they have been explored for their utility in cancer therapy. Moreover, MOFs can represent some “ideal drug delivery systems” where the organic component can represent the drug itself in a broad sense of the term (including vitamins, neurotransmitters, classical drugs, etc.). At the same time, the metal can be oligo-elements (Cu, Zn, etc.), and these components can be released by disassembling. 

Additionally, MOFs can deliver genes, making them a promising tool for various gene delivery therapies. MOFs’ high tunability and specificity recommend them for recognizing a wide range of biomolecules, making them valuable tools for diagnosing and treating various diseases and studying cancer. MOFs have also shown promise in multiple applications, including disease diagnosis and treatment, plastic waste upcycling, and blood analysis and purification. MOFs have unique properties that make them suitable for biomolecule sensing, including high surface area, tunable pore sizes, and functional groups. MOFs can be used as supports for enzymes, encased in MOFs, and protected against harsh environments, providing a stable and robust platform for enzyme-based biosensing. MOFs also possess intrinsic enzyme-like activity, known as nano-zymes, which can mimic the actions of natural enzymes. MOFs have great potential in detecting biomolecules associated with various diseases, including cardiac and circulatory diseases and cancer.

In biomedical applications beyond sensing and biosensors, MOFs have shown promise as efficient and regioselective catalysts in click reactions, efficient supports for catalytic processes, and effective antimicrobial agents. MOFs are also potentially used in 3D tissue scaffolds and as contrast agents for medical imaging. Additionally, MOFs can be used for visual glutathione detection and developing biocompatible nanomotors for cancer therapy.

Future research on MOFs in biomedical applications should address their challenges, such as precisely controlling their properties and interactions with biological systems. Researchers need to develop post-synthesis modifications to enhance the stability and biocompatibility of MOFs in biological environments. Furthermore, addressing toxicity and remaining challenges in the field is critical for fully exploring the MOF’s potential in biomedical applications.

In conclusion, MOFs represent versatile platforms for biomedical innovation with enormous potential in various applications. Future research on MOFs should focus on fully addressing the challenges and exploiting their potential in biomedical applications. With continued research and development, MOFs have the potential to revolutionize the field of medicine and improve the quality of life for many people worldwide.

## Figures and Tables

**Table 1:** The table lists MOFs by their characteristics according to CD Bioparticles, such as pore properties, including the BET-specific surface area, the pore size, the window size, and other unique features that make them suitable for medical applications. The information also includes stability in different solutions, pH responsiveness, tunable pores, capabilities for drug delivery, targeted therapy, and more.

No Crt	MOF-Type	Pore Proprieties		Specific Surface by BET [m²/g]	Characteristics	Particularities for Medical Applications
		Window Size [nm]	Pore Size [nm]			
1	ZIF-8	0.34	1.1	1500	Stable in air, aqueous, and basic solution; not stable in acid solution	pH-Responsive Drug Release [3]
2	UiO-66	0.8	1.1	1000	Stable in aqueous and acid solution	Targeted Cellular Uptake [4], high biocompatibility
3	MIL-125 (Ti)	0.6	0.8	1300	500 nm–5 µm Irregular crystal	Temperature and pH-Responsive Release [5]
4	MIL-101 (Cr)	1.2–1.6	2.9–3.4	2800–3200	Stable in air, aqueous, acid, and basic solution (pH 1–12)	Drug delivery [6]
5	MIL-101 (Fe)	1.2	2.3–2.7	2800	High surface area, tunable pores	pH-responsive drug release [7]
6	MIL-100 (Fe)	0.55–0.88	2.5–2.9	1900	Stable in air, aqueous, and acid solution	Targeted drug delivery, protein encapsulation [8]
7	MOF-74 (Mg)		1.5	900	Open metal sites, tunable porosity	Drug delivery [9]
8	ZIF-67 (Co)	0.34	1.1	1500	Stable in air, aqueous, and basic solution; not stable in acid solution	Catalysis in drug synthesis [10], targeted release [11]
9	ZIF-90 (Zn)	0.35	1.1	1200	Stable in air, aqueous, and basic solution; not stable in acid solution	Targeted drug delivery [12]
10	NU-1000 (Zr)	0.8 × 1.0	3.1 and 1.3	2200	Stable in aqueous and acid solution and organic solvents	Drug delivery [13], targeted therapy [14]
11	MOF-5	-	1.5	900	Stable in the air for several weeks	Drug Sustained Release Carrier [15]

## Data Availability

Not Applicable.
